# Can Abutment with Novel Superlattice CrN/NbN Coatings Influence Peri-Implant Tissue Health and Implant Survival Rate Compared to Machined Abutment? 6-Month Results from a Multi-Center Split-Mouth Randomized Control Trial

**DOI:** 10.3390/ma16010246

**Published:** 2022-12-27

**Authors:** Francesco Pera, Maria Menini, Mario Alovisi, Armando Crupi, Giulia Ambrogio, Sofia Asero, Carlotta Marchetti, Camilla Canepa, Laura Merlini, Paolo Pesce, Massimo Carossa

**Affiliations:** 1 Department of Surgical Sciences, C.I.R. Dental School, University of Turin, 10126 Turin, Italy; 2 Department of Surgical Sciences (DISC), University of Genoa, 16132 Genoa, Italy

**Keywords:** abutment, CrN/NbN coatings, dental implants, machined surfaces, marginal bone loss, superlattice, survival rate

## Abstract

Background: The aim of the present multi-center split-mouth randomized control trial was to investigate the effect on peri-implant tissue of abutment with chromium nitride/ niobium nitride (CrN/NbN) coatings (superlattice) compared to traditional machined surface. Methods: Two adjacent posterior implants were inserted in 20 patients. A machined abutment was randomly screwed on either the mesial or distal implant, while a superlattice abutment was screwed on the other one. Implant survival rate, peri-implant probing depth (PPD), plaque index (PI), and bleeding index (BI) were collected 6 months after surgery, while marginal bone loss (MBL) was evaluated at T0 and T6.; Results: Implant survival rate was 97.7%. A total MBL of 0.77 ± 0.50 mm was recorded for superlattice abutments, while a mean MBL of 0.79 ± 0.40 mm was recorded for the abutment with machined surface. A mean PPD of 1.3 ± 0.23 mm was recorded for the superlattice Group, and a mean PPD of 1.31 ± 0.3 was recorded for the machined surface Group. PI was of 0.55 ± 0.51 for superlattice Group and 0.57 ± 0.50 for machined Group, while BI was of 0.47 ± 0.49 for superlattice Group and of 0.46 ± 0.40 for the machined one. No statistically significant difference was highlighted between the two Groups (*p* > 0.05). Conclusions: After a 6-month observational period, no statistically significant differences were highlighted between superlattice abutment and traditional machined abutment. Further in vitro studies as well as clinical research with longer follow-ups are required to better investigate the surface properties of the novel abutments’ superlattice coating and its effect on the oral tissues.

## 1. Introduction

Since the first introduction of titanium implants, today, implant prosthodontics have been considered the standard treatment to rehabilitate edentulous patients with a fixed prosthesis [1,2]. In order to provide long-term survival and success rates, two key factors are fundamental [3,4,5]; first is the achievement of a correct primary osseointegration; and second is the maintenance of the peri-implant hard and soft tissue health over the years [6]. In the last decades, research has focused on the investigation of the effect of different implant surfaces on the osteoblast response in order to obtain a correct and predictable osseointegration [7,8]. As highlighted in the systematic review by Wennerberg and Alberktsson [9], rougher surfaces demonstrated the capability of enhancing a stronger bone response and osseointegration, even after having been exposed to bacteria contamination [10]. Based on these results, rough implant surfaces are now more commonly preferred in contrast to the smooth surfaces. On the other hand, in recent years research has started to investigate whether the surface characteristics of the abutment components can play a role in the long-term maintenance of the peri-implant tissue health.

This prosthetic component, which is routinely adopted to avoid excessive loads on the peri-implant bone [11] and to move the prosthetic platform far from the implant head [12,13], is also the transmucosal component directly in contact with soft tissue during the healing process and in the long-term period. Different studies demonstrated how the soft tissue healing process around the abutment is a fundamental factor to obtain thick and stable soft tissue sealing around the implant. [14,15]. Therefore, different abutment materials and surface treatments have been proposed and investigated to enhance the fibroblast proliferation and attachment around the surface [16,17,18]. Among others, anodized [19], machined [20] and polished [21] surfaces are the most common surface treatments adopted during the manufacturing process, while ultraviolet light [22] and plasma of argon [23,24] were proposed to decontaminate the surface and increase the hydrophilicity of the surfaces after the manufacturing process. In a recent systematic review of in vitro studies, Corvino et al. [25] analyzed the cellular response of fibroblasts on different abutment materials and surface modifications. The authors’ findings highlighted how different treatments are capable of influencing the fibroblast response, however, a variety of studies and results were found. In a systematic review, Carossa et al. [26] analyzed the effect of Plasma of Argon treatment on the cellular activity around titanium. The authors highlighted how the in vivo studies are limited and the in vitro applications seem to show positive results during the first few hours, whereas the duration of the effect remain unclear. Pesce et al. [27] performed a systematic review of histological findings on the effect of modified titanium abutment on peri-implant soft tissue behavior. The authors found limited evidence and their result showed an enhanced connected fiber attachment for the abutment with modified surfaces. However, the result of the study also showed how the modified surfaces may be prone to a higher risk of tissue inflammation over the time. From the analysis of the data currently available, it can be therefore assumed that the abutment surface characteristic plays a role in influencing the soft tissue response and the healing process, which usually happens within the first 6 months post-surgery. However, the data is controversial and a final consensus of what surface is preferable is currently absent. Furthermore, the majority of the research is limited to in vitro studies, while in vivo studies on the topic are currently lacking.

**Figure 1:**
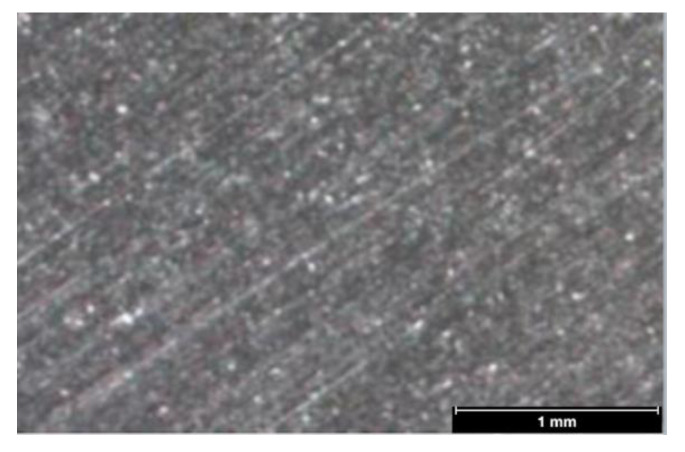
Microscopic image showing surface characteristic of the CrN/NbN coating manufactured by Lafer S.P.A., Piacenza, PC, Italy.

Recently a novel chromium nitride/niobium nitride (CrN/NbN) coating, defined as “superlattice” coating [23], which is obtained during the manufacturing process through the deposit of 1200 alternated layers of CrN and NbN (Figure 1), was made available for dentistry application (CE N°1935/2004).

Currently, the only available data from the literature is from orthopedic implant applications [28,29,30]. To the authors knowledge, no data is currently available on its application for dental implant purposes.

Therefore, the aim of the present multi-center split-mouth randomized control trial was to test whether titanium abutment with CrN/NbN coatings (superlattice) can influence the peri-implant tissue behavior compared to the traditional machined abutment after a 6-month observational period.

The null hypothesis was that there is no clinical difference between the two abutments.

## 2. Materials and Methods

The present research was performed according to the principles outlined in the Declaration of Helsinki on experimentation involving human subjects. All patients enrolled in the study were thoroughly informed about the research purpose and signed an informed consent form prior to undergoing the procedures. The study protocol and the research were approved by the local ethical committee of the University of Turin (Protocol N° 0123972) and of the University of Genoa (CERA 2020/29).

The present study was performed following the Strengthening the Reporting of Observational Studies in Epidemiology (STROBE) guidelines.

### 2.1. Study Design

The present study was designed as a multi-center split-mouth randomized control trial. Two different centers participated in the study: (1) the Prosthodontics and Implant Department of the C.I.R. Dental School, Department of Surgical Sciences, University of Turin, Italy; and (2) the Division of Prosthodontics and Implant Prosthodontics, Department of Surgical Sciences, University of Genova, Italy. A multi-center design was adopted to enroll and compare a larger number of patients from two different Italian regions (Turin-Pidmonte and Genoa-Liguria).

**Figure 2:**
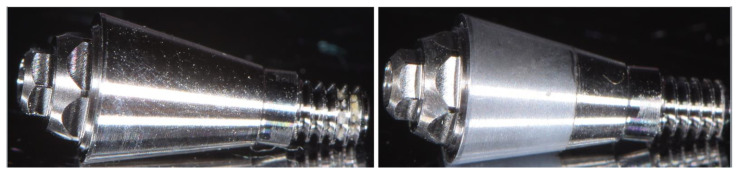
Image showing the macroscopic aspects of the two abutments used in the present study. **Left**: machined abutment; **Right**: superlattice abutment.

A split-mouth design was adopted to decrease the risk of bias due to the possible different uncontrolled variables between patients. It was performed by the insertion of 1 abutment with superlattice and one traditional machined abutment (Figure 2) in the same patient on two adjacent different implants.

The abutments were randomly assigned to the mesial or distal implant through a computer-generated random sequence of numbers (SPSS 24.0; SPSS Inc., Chicago, IL, USA).

All the implants were inserted by the same two experienced clinicians (one per each center) who were aware of the abutment they were using.

All the follow-up measurements were performed by two experienced calibrated operators at each center who were blinded regarding the treatment procedures of the abutments.

Cohen’s kappa statistic was used to calculate observer agreement and sample size calculation was performed with G*Power 3.1 (Kiel University, Kiel, Germany).

### 2.2. Patients Selection

From April 2022, the waiting list of the Prosthodontics and Implant Department of the C.I.R. Dental School, Department of Surgical Sciences, University of Turin and of the Division of Prosthodontics and Implant Prosthodontics, Department of Surgical Sciences, University of Genova were sought to recall patients requiring multi-unit implant rehabilitations.

The inclusion criteria were: edentulous patients in the posterior area requiring implant rehabilitation with two adjacent implants; availability of adequate bone at both the vertical and horizontal dimensions; absence of systemic conditions; and implant insertion torque > 35 Newton.

Exclusion criteria were: presence of systemic complications and history of bisphosphonate drug therapy; signs of parafunctional activities; inability to come at the control visit; requirement for regenerative procedures; and smoking patients.

Patients underwent clinical examination and periapical X-rays and cone beam computed tomography (CBCT) were acquired to evaluate the availability of bone at both the horizontal and vertical dimension.

Patients who met the inclusion/exclusion criteria were enrolled in the study and surgery appointments were planned.

### 2.3. Surgical Appointments

As pre-operative antibiotic therapy, amoxicillin 875 mg + clavulanic acid 125 mg every 12 h for 6 days beginning the day before the surgery was used [31,32]. The mouth was rinsed for one minute with chlorhexidine 0.2% (Curasept S.p.A., Saronno, Italy) just before surgery.

**Figure 3:**
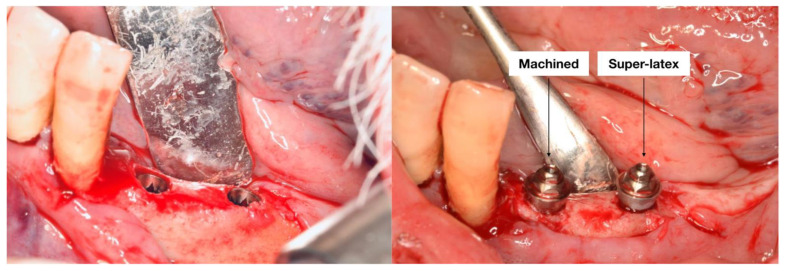
Figure showing an example of the surgery procedure (**left**) and of the split-mouth insertion of the MUAs (**right**).

Local anesthesia was given with 4% articaine and 1:100,000 adrenaline (Alfacaina SP; Dentsply Italy, Rome, Italy). A full thickness mucoperiostal flap was elevated. The osteotomy was conducted following the manufacturer’s instruction. Two adjacent bone level implants were then inserted in each patient (Shard implant, Mech and Human, Grisignano di Zocco, VI, Italy) and two multi-unit abutments (MUA) (Straight M.U.A., Mech and Human, Grisignano di Zocco, VI, Italy) were then screwed into the implants. Following the split-mouth design of the study, in the same patient one abutment had a traditional machined surface, while the other one had a superlattice surface treatment (Figure 3).

MUAs were randomly assigned to the mesial or distal implant through a computer-generated random sequence of numbers (SPSS 24.0; SPSS Inc., Chicago, IL, USA). Then, two healing cups (PMCGU05000000, Mech and Human, Grisignano di Zocco, VI, Italy) were screwed on the M.U.A. and the full thickness flap was sutured using silk multi-filament sutures (PERMA-HAND SILK 4-0; Ethicon, Somerville, NJ, USA) with interrupted sutures at both the mesial and distal side of each implant.

Periapical X-rays were acquired.

Patients were then instructed with postoperative recommendations including a 0.2% chlorhexidine digluconate rinse (Corsodyl, GlaxoSmithKline, Verona, Italy) twice a day for two weeks, soft diet, and hygienic instructions.

Patients returned one week post-surgery for a checkup and suture removal.

**Figure 4:**
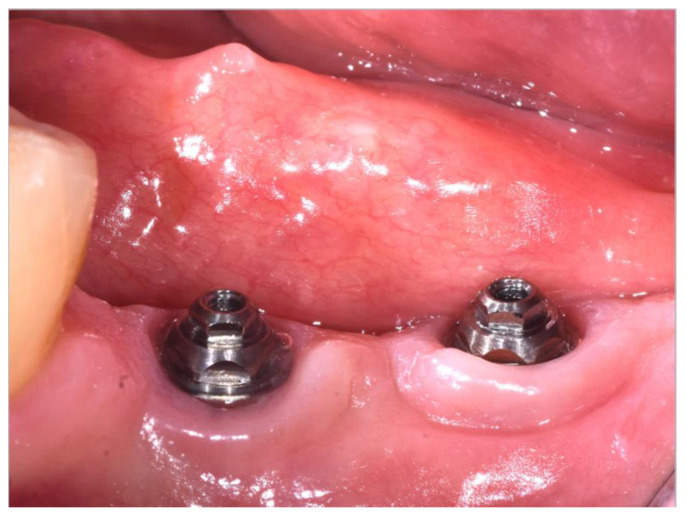
Clinical image showing the healing after 3-month post surgery. **Left**: machined abutment; **Right**: superlattice abutment.

Patients returned to the clinic three months after surgery (Figure 4) and periapical X-rays were acquired.

Healing cups were removed and an open tray impression was made using two transfers M.U.A. (PMTK105000000, Mech and Human, Grisignano di Zocco, VI, Italy)

After the laboratory process, two splinted crowns were made of metal-composite. The prosthesis was delivered and occlusion was carefully checked.

Checkups were planned at 6 months post-surgery.

### 2.4. Clinical Outcomes

**Figure 5:**
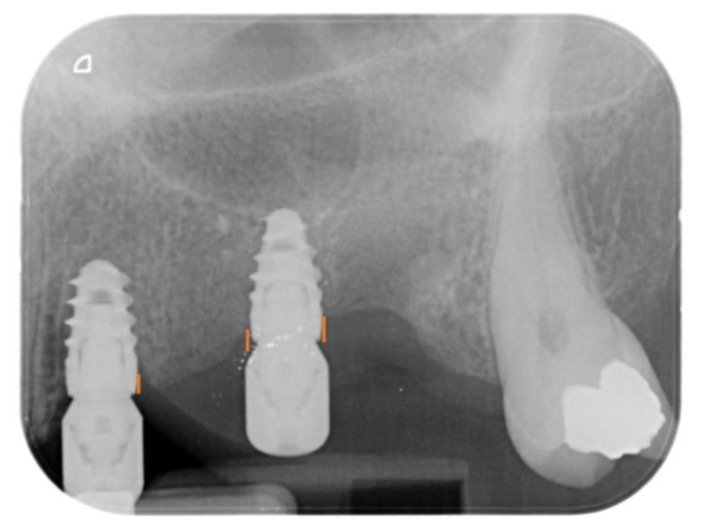
Periapical X-rays showing the distance between the implant-abutment interface and the most coronal aspect of the bone. **Left implant**: implant with machined abutment; **Right implant**: implant with superlattice abutment.

The primary clinical outcomes were implant survival and prosthetic complications. Secondary clinical outcomes were:

- Marginal bone loss (MBL), evaluated at both the mesial and distal aspects of the implants immediately after surgery (T0) and at 6 (T6) months after surgery. MBL was evaluated on intraoral apical X-rays by measuring the distance between the implant-abutment interface and the most coronal aspect of the bone (Figure 5) using Imagej software. The known length of the implant was used to set the mm scale. The difference between T6 and T0 resulted in the MBL.
- Peri-implant soft tissue parameters, including peri-implant probing depth (PPD), plaque index (PI), and bleeding index (BI). PPD was evaluated using a HuFriedy PCPUNC 15 probe (HuFriedy, Chicago, IL, USA) at the mesio-buccal, buccal, disto-buccal, mesio-lingual, lingual, and disto-lingual aspect of each implant. PI and BI were evaluated as number of surfaces (the mesio-buccal, buccal, disto-buccal, mesio-lingual, lingual, and disto-lingual) presenting plaque or bleeding on probing. All the parameters were evaluated at T6.

### 2.5. Statistical Analysis

Univariate linear regression models were used to investigate a significant correlation between the machined abutment and the superlattice abutment with MBL and PPD. Results with *p* < 0.05 were considered significant. All the assessments were performed using SPSS IBM (version 25)

## 3. Results

In total, 20 patients (n = 20) met the inclusion criteria and were enrolled and treated, for a total implant number of 40 (n = 40); a total machined abutment number of 20 (n = 20); and a total superlattice abutment of 20 (n = 20). A total of 10 patients were treated at the University of Turin and 10 patients were treated at the University of Genova. Relevant data regarding patient population and implant characteristics is summarized in Table 1.

One implant with superlattice abutment failed and was removed 6 months after the surgery. The implant was inserted in the left first molar position. No parafunctional activity or risk factors were detected. The non-ossointegration was noticed during peri-implant soft tissue evaluation when a movement of the implant was detected. The implant was easily extracted and no tissue inflammation or pain were detected. In absence to other factor associated, the reason of the failure was attributed to a lack of osseointegration. The overall survival rate was 97.7% (n = 39). Implants with machined abutments presented a 100% survival rate (n = 20), while implants with superlattice abutments presented a 95% survival rate (n = 19). No prosthetic complications were observed for the implants with survival.

Excellent intra-observer (kappa values of 0.78 and 0.80) and inter-observer (a kappa value of 0.80) agreements were recorded in this study. A total MBL of 0.78 ± 0.45 mm (mesial 0.77 ± 0.44 mm and distal 0.78 ± 0.56 mm) was recorded. By dividing for abutment surface treatment, a total MBL of 0.77 ± 0.50 mm (mesial 0.76 ± 0.49 mm and distal 0.77 ± 0.50 mm) was recorded for superlattice abutments, while a mean MBL of 0.79 ± 0.40 mm (mesial 0.78 ± 0.38 mm and distal 0.79 ± 0.42 mm) was recorded for the abutment with machined surface.

In general, healthy and stable soft tissue were observed for both of the groups. A mean PPD of 1.3 ± 0.23 mm was recorded for superlattice abutments, and a mean PPD of 1.31 ± 0.3 was recorded for the abutment with machined surface. PI was of 0.55 ± 0.51 for superlattice Group and 0.57 ± 0.50 for machined Group, while BI was of 0.47 ± 0.49 for superlattice Group and of 0.46 ± 0.40 for the machined one.

The available sample size made it possible to reach a power of the study higher than 80%. No statistically significant difference (*p* > 0.05) was highlighted between the machined and superlattice groups in regards to MBL and PPD (Table 2).

## 4. Discussion

The aim of the present multi-center split-mouth randomized control trial was to test whether titanium abutment with superlattice can influence the peri-implant tissue behavior compared to traditional machined abutment. In total, 40 implants (n = 40) were inserted in 20 patients (n = 20). In each patient, a machined abutment was randomly screwed into the implant during the surgery on either the mesial or distal implant, while a superlattice abutment was screwed on the other implant (split-mouth design). Therefore, a sample size of n = 20 was obtained for each group. After the 6-month follow-up, 1 implant with superlattice abutment failed, resulting in an implant survival rate of 95% (n = 19), while all the implants with machined abutment were successfully integrated (n = 20, survival rate = 100%). At T6, implant with machined abutment presented a MBL of 0.79 ± 0.40 mm (mesial 0.78 ± 0.38 mm and distal 0.79 ± 0.42 mm), while MBL for implants with superlattice abutment was 0.77 ± 0.50 mm (mesial 0.76 ± 0.49 mm and distal 0.77 ± 0.50 mm). PPD of 1.3 ± 0.23 mm and of 1.31 ± 0.3 was recorded respectively for superlattice and machined groups. The statistical analysis did not reveal any statistically significant differences between the 2 group in any of the parameters analyzed. Therefore, the null hypothesis was accepted.

In the present study, a modern implant presenting internal conical connection allowing platform switching [33] was adopted. The results observed in the present study regarding implant survival rate and MBL of posterior implant supporting multi-unit fixed prosthesis (ISMFP) are in agreement with the data described in the literature with similar observational periods and different implants and techniques. Menini et al. [34] reported the outcome of 38 ISMFP placed either with one-stage or two-stage technique. The authors findings showed a survival and success rate of 100% and a MBL of 0.46 ± 0.41 (two-stage) and 0.45 ± 0.38 (one-stage) mm after a 1 year observational period. In agreement, Abi-Aad et al. [35] reported the outcomes of 153 ISMFP, placed either following an immediate or delayed loading protocol, and found a MBL of 0.42 mm for the immediately loaded group and 0.46 mm for the conventionally loaded implants at the 1 year follow-up.

In regards to the abutment surface treatment, different article investigated the properties of CrN/NbN coatings (superlattice) from a materials and engineering perspectives [36,37,38,39]. This superlattice structure is obtained by combining Cr, that present excellent tribological properties, and Nb, which represents one of the metals with the highest chemical stability. As a result, the main reported advantages are optimal corrosion and abrasion resistance; excellent biocompatibility; and increased adhesion to metals [28]. Indeed, it presents fine-scale surface porosity which results in micro-structural improvement and better corrosion performance compared to CrN alone [36]. From a dentistry point of view, to the author’s knowledge, no data is currently available on its usage for dental application and the only available data on medical implants are from orthopedic implants application [28,29,30]. Following the manufacturer’s description, the physical properties of the coating applied on the abutment are: coating thickness 2–6 μm; coating hardness: 2500 ± 200 HV; Roughness Ra: 0.08–0.10 μm; friction coefficient: 0.3–0.4.

Following the 6-month result of the present study, a slightly less non-significant MBL was noticed for the superlattice abutment compared to the machined one. Therefore, data from the present study indicates promising results and superlattice treatment seems to be a valid alternative to the traditional machined surface. However, it must be highlighted that the observational period was limited and further in vitro and clinical studies with longer follow-up are required to confirm the result. Furthermore, no comparison between the variables considered was performed at the 3-month follow-up (implant loading time) and no trial registration was performed for the present research, representing additional limitations of the study.

The 6-month cut off was decided for the present article in agreement with different systematic reviews on histological findings [27,40]. Indeed, assuming that the abutment material is capable of influencing the healing process, 6-months were reported to be enough to evaluate the first healing of the soft tissues. The results obtained from the first 6-month follow-up encourage the research to continue in order to evaluate the long-term effect that these components may have on the peri-implant tissue behavior.

To date, results from in vivo studies investigating the effect of different abutment surface treatments on the peri-implant tissue behavior are controversial. In a randomized control trial, Canullo et al. investigated the effect of Plasma of Argon cleaning on abutment surfaces. After an observational period of 18 [41] and 24 [42] months, the authors found positive results in terms of hard tissue level changes for the abutment cleaned with plasma of argon treatment. Abrahamsson et al. [43] compared the effects of turned and acid-etched titanium abutments on the clinical soft tissue adhesion. Results from the study showed that the soft tissue response was not significantly affected by the surface roughness. On the contrary, results from the study on canine mandible of Hermann et al. [44] were found in disagreement with the ones from Abrahamsson et al. [43] The authors compared 30 machined collars and 30 acid-etched collars and found a significantly less MBL for the acid-etched group. Similarly, they were also shown in the randomized control trial by Schwarz et al. [45] who investigated abutment with 3 different surface treatments. In agreement with Hermann et al. [44] and in disagreement with Abrahamsson et al. [43], the authors found an influence of the surface roughness on the soft tissue response, and demonstrated how moderately rough surfaces can be beneficial for soft tissue adhesion.

Furthermore, a recent systematic review and meta-analysis by Sanz-Martín et al. [46] analyzed the effects of modified abutment characteristics on peri-implant soft tissue health. The authors analyzed 19 articles with a minimum observational period of 6 months and found how zirconia abutment systemically presents less bleeding on probing compared to traditional titanium abutment.

Research on different abutment surfaces has become important also in light of the results from a recent systematic review from Tallarico et al. [47] who demonstrated how the implant-abutment interface can be prone to bacteria contamination and, thus can play a role in the onset of peri-implantitis. To overcome this problem, Carossa et al. [48] suggested a possible application of tissue level implants without the usage of abutments. However, scientific evidence on the topic is lacking and the current available data indicates the absence of the abutment as a possible risk factor increasing MBL.

In conclusion, data on the abutment characteristics from the literature is currently controversial and research on the abutment characteristics remains a current and open topic [49,50,51].

The present study represents the first report on the usage of superlattice as a coating for implant abutment surfaces. Data from the present study seems promising for its usage in dental applications. However, further studies with longer follow up are required to better understand the long-term clinical outcomes of using this surface coating as transmucosal abutment.

## 5. Conclusions

Within the limitations of the present study, both the abutments showed acceptable clinical outcomes. No statistically significant differences were highlighted between superlattice and traditional machined abutment after an observational period of 6 months. Further in vitro studies, as well as clinical research with longer follow-ups are required to better investigate the surface properties of the novel abutments’ superlattice coating and its effect on the oral tissues.

## Figures and Tables

**Table 1:** Relevant data regarding patients’ population and implant characteristics.

Characteristics	Patients (n = 20)
Age (mean ± SD)	40 ± 13
Male	35% (n = 7)
Female	65% (n = 13)
	**Implants (n = 40)**
Length 13 mm	7.5% (n =3)
Length 10 mm	72.5% (n = 29)
Length 8.5 mm	15% (n = 6)
Length (mm, mean ± SD)	10 ± 0.45
Diameter 4.3 mm	100% (n = 40)

**Table 2:** Univariate statistical analysis regarding MBL and PPD at 6 months follow-up (T6).

Variable	Coefficients	*p* Value
Mesial MBL	−0.133	0.419
Distal MBL	−0.092	0.579
PPD	0.036	0.826

## Data Availability

The datasets generated during and/or analyzed during the current study are available from the corresponding author on reasonable request.
